# Assessment and counseling to get the best efficiency and effectiveness of the assistive technology (MATCH): Study protocol

**DOI:** 10.1371/journal.pone.0265466

**Published:** 2022-03-16

**Authors:** Thais Pousada García, Betania Groba Gonzalez, Laura Nieto-Riveiro, Nereida Canosa Domínguez, Saturnino Maldonado-Bascón, Roberto J. López-Sastre, Soraya Pacheco DaCosta, Isabel González-Gómez, Alberto J. Molina-Cantero, Javier Pereira Loureiro

**Affiliations:** 1 CITIC (Centre for Information and Communications Technology Research), TALIONIS Research Group, Universidade da Coruña, A Coruña, Spain; 2 GRAM, Department of Signal Theory and Communications, University of Alcalá, Madrid, Spain; 3 FINEMEV, Department of Nursing and Physiotherapy, University of Alcalá, Madrid, Spain; 4 Department of Electronic technology, E.T.S.I. Informatics. Universidad de Sevilla, Sevilla, Spain; Universitat de Valencia, SPAIN

## Abstract

**Aims:**

To determine the psychosocial impact of assistive technology(AT) based on robotics and artificial intelligence in the life of people with disabilities.

**Background:**

The best match between any person with disabilities and its AT only can be gotten through a complete assessment and monitoring of his/her needs, abilities, priorities, difficulties and limitations. Without this analysis, it’s possible that the device won’t meet the individual’s expectations.

Therefore, it is important that any project focused on the development of innovating AT for people with disabilities includes the perspective of outcome measures as an important phase of the research. In this sense, the integration of the assessment, implementation process and outcome measures is crucial to guarantee the transferability for the project findings and to get the perspective from the final user.

**Methods:**

Pilot study, with prospective, longitudinal and analytical cohort. The study lasts from July 2020 until April 2023. The sample is formed by people with disabilities, ages from 2–21, that will participate from the first stage of the process (initial assessment of their abilities and needs) to the final application of outcome measures instruments (with a complete implication during the test of technology).

**Discussion:**

Only with the active participation of the person is possible to carry out a user-centered approach. This fact will allow us to define and generate technological solutions that really adjust to the expectations, needs and priorities of the people with disabilities, avoiding the AT from being abandoned, with the consequent health and social spending.

**Trial registration:**

Clinical Trials ID: NCT04723784; https://clinicaltrials.gov/.

## Introduction

The present paper offers the description of a subproject of a global and complete coordinated project, in which main participants are the University of Alcalá (UAH), the University of Sevilla (US), and the University of A Coruña (UDC), which title is “Artificial Intelligence and Robotic Assistive Technology devices for Disabled People (AIR4DP)”.

Assistive Technology (AT) is an umbrella concept that refers to devices with different levels of technological complexity. AT improves the functional capacity in different areas of a person’s life. Some of these areas have been described in [1] as a) eating (e.g., adapted cutlery, dishes with flange); b) mobility (e.g., powered wheelchairs, walkers, canes); c) communication (e.g., augmentative and alternative communication systems); d) access to the computer (e.g., screen readers, adaptations for keyboards, push buttons, computer systems, switch); e) housing; f) transport; and g) leisure or sports. Therefore, AT are devices that facilitate and/or allow the development of activities of the daily living of a person with a disability, providing resources to promote independence and quality of life (QoL).

However, despite the clear benefits of the use of assistive technology devices, it has also been described some associated problems. The most common ones can be grouped into several categories: lack of information about available AT, economic factors related to the high price of products; access to assistive devices and technology; as well as factors related to the guarantee, maintenance and insurance of the AT [2–4].

One of the most frequent and influential factors during the process of prescription and subsequent use of the AT is the lack of matching between the person and the recommended AT [5,6]. This problem may lead to the abandonment of the AT. The non-use of devices has clear causes and obvious consequences not only on the quality of life and the independence of the person with a disability but also cost-benefit effects and negative impact on the health system [7]. To achieve an increase in the successful use of AT and, therefore, to reduce the probability that these solutions are abandoned or that they do not fulfill their function, it is essential to carry out an adequate assessment of the user’s needs with reliable measurement tools to improve decision making for the prescription and adaptations of AT.

The outcome measure is a set of considerations and tools to determine if a service, product or device allows achieving the goals for which it has been created, under criteria of efficacy, and effectiveness. In other words, outcome measure implicates the evaluation process in the provisioning service, and this is designed to quantify and to establish a baseline on something that works (its effectiveness), the group of persons on which it works, and the level of economic efficiency it provides [8]. So, professionals prescribing AT, including nurses, have to take into account the need for applying outcome measures to improve their intervention, with a process evidence-based.

World Health Organization (WHO) General Assembly published in 2018 a resolution on the importance of AT and the related services globally (including assessment), their importance on the health system and supported position papers. The main considerations that have to be taken into account by the governments in the provision of assistive technology to all citizens are related to the statements that emerged from the Global Research, Innovation, and Education on Assistive Technology (GREAT) Summit: Personal, Products, Provision, and Policy [9].

To give support to the professionals, to improve the services of AT and to benefit the final users of those devices, several models of outcome measure have been proposed [6,10]. Those models indicate the main factors to consider in the process of matching the AT with the person: The functional problems that the AT intends to solve; the characteristics of final users and their needs and priorities; the characteristics of the device; the context in which the AT is applied or used; the expected changes in the state of a user and its context as the results of using AT; and the impact of AT devices on the individual’s participation.

A revision done by the Assistive Technology Outcomes Measurement System (ATOMs) Project identified 22 models of outcome measures related to AT, and 14 tools or instruments to assess the results of that types of devices, such as the psychosocial impact of AT on the quality of life, satisfaction with the device or level of matching between person and technology [11]. In Spain, the research related to outcome measures in AT is deficient, because there are not many specific measurement instruments that are validated for the Spanish population. The application of these tools has been published only in a few research projects [12–16].

This paper is focused on the implementation of outcome measurement tools to improve the efficacy, effectiveness, and real utility of the developed robotic AT, based on Artificial Intelligent (AI). On the other hand, it is also important to assess the functional impact the AT has on users’ participation in their social environment.

AIR4DP project consists of the three sub-projects:

- *Artificial Intelligence and Robotic Mobile Platforms to Improve Disabled People Independence (AIRPLANE)*: It is presented by the University of Alcalá (UAH). It aims to offer to the AIR4DP project advanced novel mobile robotic platforms with advanced perception, interaction and navigation capabilities, able to stimulate and monitor the improvement of the independence of people with disabilities. The robotic platforms can autonomously navigate the environment, (e.g., the house of the user with disability), track the activity of the users, anticipate dangerous situations (e.g. a fall), and help with the sequencing of daily living activities.
- *Augmentative Affective Interface (AAI)*: Searches for a better human-robot experience by including remote monitoring and local wearable sensors to collect the subject’s physiological data for both emotional state detection and activity recognition. Namely, AAI looks for improving the quality of life of people with disabilities by adapting and promoting physical activity autonomously but in a controlled scenario. The scientific objective of this subproject is to develop new wearable technical solutions for monitoring physiological aspects to help the detection of emotional states and user activities
- *Assessment and counseling to get the best efficiency and effectiveness of the Assistive Technology (MATCH)*: This project is the complement to get the best evidence about the impact of assistive technology based on AI in the life of a person with a disability. The perfect match between person and technology can only be achieved through a complete assessment and monitoring of the capabilities, needs, priorities, difficulties, and limitations that a person has in his/her life. Without these analyses, there is a risk that the technology does not adapt to the expectations of the user. Then, the person in a short time abandons the assistive technology resulting in unnecessary expenditure of resources. So, it is relevant that within the coordinated project, a team with experience in evaluation, design, and outcome measures of assistive technology for people with disabilities is integrated into the consortium.

The consortium formed will allow covering from the implementation of intelligent mobile platforms to improve independence (AIRPLANE) and multi-modal wearable sensor solutions for monitoring people (AAI), to the assessment, from a health point of view, of the impact of these aids on people with disabilities (MATCH), to improve their Quality of Life (QoL).

In this Study Protocol, the justification and the development of the subproject MATCH is explained. It will be coordinated by the University of A Coruña, with the collaboration of the research groups from UAH and US, and its performance implies the participation of people with disabilities.

### Aims

The general objectives are: (1) to get the best evidence about the impact of robotic assistive technology in the life of people with disabilities; (2) to promote the best match between the user and this assistive technology.

To complete, a few secondary goals have been addressed:

- To assess the functional skills and abilities of people with disabilities, and to identify their needs to get independence in mobility and activities of daily living.
- To design and create the best technology solutions for each person (the AT that adapts intelligently to the demands of each participant), meeting with the principles of “design for all”, and promoting the involvement of the final user.
- To validate the assistive technology’s solutions designed and prototyped to increase the levels of participation in daily living activities and mobility for people with disabilities.
- To validate a model of outcome measures in the field of robotic AT to increase the efficiency of selecting and prescribing these products.

## Materials and methods

The design of this research corresponds with a pilot study, with prospective, longitudinal and analytical cohorts.

The whole project has a duration of 36 months, lasting from July 2020 until April 2023.

The development of the fieldwork of research is being developed with the collaboration of rehabilitation and education centers of people with disabilities (they are presented in the “environment of the study”). Each university has an agreement with centers of different non-profit organizations (NGOs) attending to people with disabilities that will participate in the study.

### Sample and participants

The selection of the sample is limited to the users of the centers where the project will be implemented. The participants will be those that meet the inclusion criteria and not with the exclusion ones. They will be given their informed consent to participate in the research.

The inclusion criteria are the following:

- People with recognized disability, derived by a disease or a permanent health status (with the respective certificate, according to the Spanish law [17].
- Age between 2 and 21 years old. This criterion is conditioned by the collaborators’ centers with the project. The special schools and educational centers are offering services to people from 2 to 21 years old, that is the maximum age allowed for attending this type of special education.
- To receive services from one of the collaboration centers.
- To have a level of functional independent moderate–low (assessed by the Functional Independence Measure (FIM) for adults or Wee FIM for children younger than 7, as appropriate).
- To have a level of functional skills–mobility moderate–low (assessed by the Spanish version of Pediatric of Disability Inventory–PEDI).

The exclusion criteria are: (1) To have any health status that is incompatible with the use of assistive technology designed and prototyped in the project; (2) to have very limited cognitive skills which make them difficult to follow the instructions to use the AT properly. The cognitive skills are determined by the application of the Cognitive Section from the Functional Independence Measure. If the score obtained in this section is lower than 20 (over 35 points) it will be an exclusion criterion. (3) Not to have adequate human support or caregivers to make use of AT.

The research group has also fixed criteria for the participants, in the case of leaving the study: Voluntary abandonment of the research and/or to finish the relationship with the collaborator center where the research project is carrying on.

#### Selection of participants

All persons attending the collaborator centers that meet inclusion criteria will be invited to participate in the research. The selection of participants will be done by each research group (UAH, US and UDC), through their contact with collaboration centers (there are specified below). In all cases, an information letter with the whole information of the project will be provided to the managers or directors of these centers, to get the authorization to develop the research project.

This project is designed for the purpose of a pilot study, with participation conditioned by available resources. Thus, the sample size of the study will be determined by the number of assistive technology devices generated and that can be donated to the collaborating centers so that they can be tested and tested by their users and professionals.

According to the bibliography consulted, it is recommended to include between 30 and 50 participants to carry out the pilot studies [18]. In this case, the participation of 5 to 10 users by center is expected, who respond to the specified inclusion criteria, that is, they have the attributes that are to be measured in the target population.

### Study setting

The project will be done by researchers from the Universities of Alcalá (UAH), Sevilla (US) and A Coruña (UDC). So, the context of the study is restricted to those locations in Spain.

In the case of UAH, the context is located in Alcalá de Henares and also a few districts of Madrid. The centers of people with disabilities that will be able to participate are: Centro de Atención Integral SAIDI-APHISA, Fundación ASTOR, Public Nursery and Primary School (CEIP) Luis Vives, Public Special Education School Pablo Picasso and ATENPACE. The US will carry out the project in the city of Sevilla and its metropolitan area. The collaborator centers will be: Association of People with Cerebral Palsy (ASPACE Sevilla) and Special Education School Directora Mercedes Sanromá. In the case of UDC, its context of study includes the metropolitan area of A Coruña, with the collaboration of the Association of People with Cerebral Palsy (ASPACE Coruña).

The research group is formed by researchers from the three universities, and also by professionals of those collaborator centers.

### Data collection

The study variables and the correspondent measure instruments are specified in Table 1. The use and the application of each instrument are conditioned by the stage of the intervention process, which is defined and described in the next section. All instruments have been translated and validated in the Spanish context. Access to the clinic history of the participants won’t be required for the performance of this project. Nevertheless, all obtained results will be codified and managed through RedCAP Database, which is a secure web application for building and managing online surveys and databases [19].

**Table 1:** Study variables and measure instruments.

Variable	Measure instrument		Components of variable
Socio-demographic characteristics	Specific questionnaire		Age Gender Marital Status Co-living with City of residence Level of Studies Main occupation Center/Association Diagnosis/Functional Status
Level of Independence in the performance for activities of daily living	Pediatric population: *Participants younger than 7 years old*	Wee FIM: Functional Independence Measure	Motor Skills: eating, grooming, bathing, dressing–upper body, dressing–lower body, toileting, bladder management, bowel management, and transfers bed/chair/wheelchair, toilet, tub/shower, walk, stairs. Cognitive Skills: auditory comprehension, verbal expression, social interaction, problem-solving, and memory
	Adult population: *Participants older than 7*	FIM: Functional Independence Measure	
**Personal mobility** (capacity for indoor and outdoor mobility, and risk of falls) *(to be filled by professional)*	Pediatric population: *according to the age of application*	Pediatric Balance Scale *(from 5 to 15 years old)* Gross Motor Function Measure *(younger than 16 with cerebral palsy)*	Skills for walking, balance and risk of falls. *To select the measure that corresponds*: *The GMFM is specific for children and adolescents with Cerebral Palsy*, *younger than 16*.
	Adult population *(older than 16)*	Test up & go 10-meter walk test 6-minute walk	
Level of satisfaction in different domains	**Matching Person and Technology Model (MPT):** **Assistive Technology Device Predisposition Assessment** (ATD PA)–Section B *(to be filled by the user)* *The questionnaire is used as a satisfaction subjective measure and it allows prioritizing those aspects of his/her life where the intervention is more desired (12 items)*		Able to go wherever desired, Self-care and domestic tasks, Interpersonal interactions and relationships, Close, intimate relationships, Educational attainment, Work and employment status/potential, Participation in desired community, social and civic activities, Autonomy and self-determination, Fitting in, belonging, feeling connected, Emotional well-being, Physical comfort & well-being, Overall health.
Priorities and needs of the person in different vital domains.	**Matching Person and Technology (MPT):** **Initial Worksheet** *(only it needs to fill those domains in which the person detects any difficulty*, *highlighting the strengths and the goals of the user in each domain)*		Thinking, Understanding, and Remembering, Seeing, Hearing, Speech and Communication, Reading and Writing, Learning, Mobility, Dexterity/Hand Use, Self-care/Health Maintenance, Domestic Life and Household Activities, Interpersonal Interactions, Employment, Recreation, Leisure and Social/Community Life
Psychosocial impact of the assistive device on the life of the person	**Psychosocial Impact of Assistive Scale (PIADS)** *(to be filled after two months from the first use of the device)*		Competence Adaptability Self-esteem
Level of matching between person and technology	**Matching Person and Technology** **Assistive Technology Device Predisposition Assessment** (ATD PA)–Device form *(to be filled after two months from the first use of the device)*		12 items related to the level of adaptation of the assistive technology in different situations and contexts. The score of each item varies from a Likert scale of 1 (rarely) to 5 (always).

**Fig 1:**
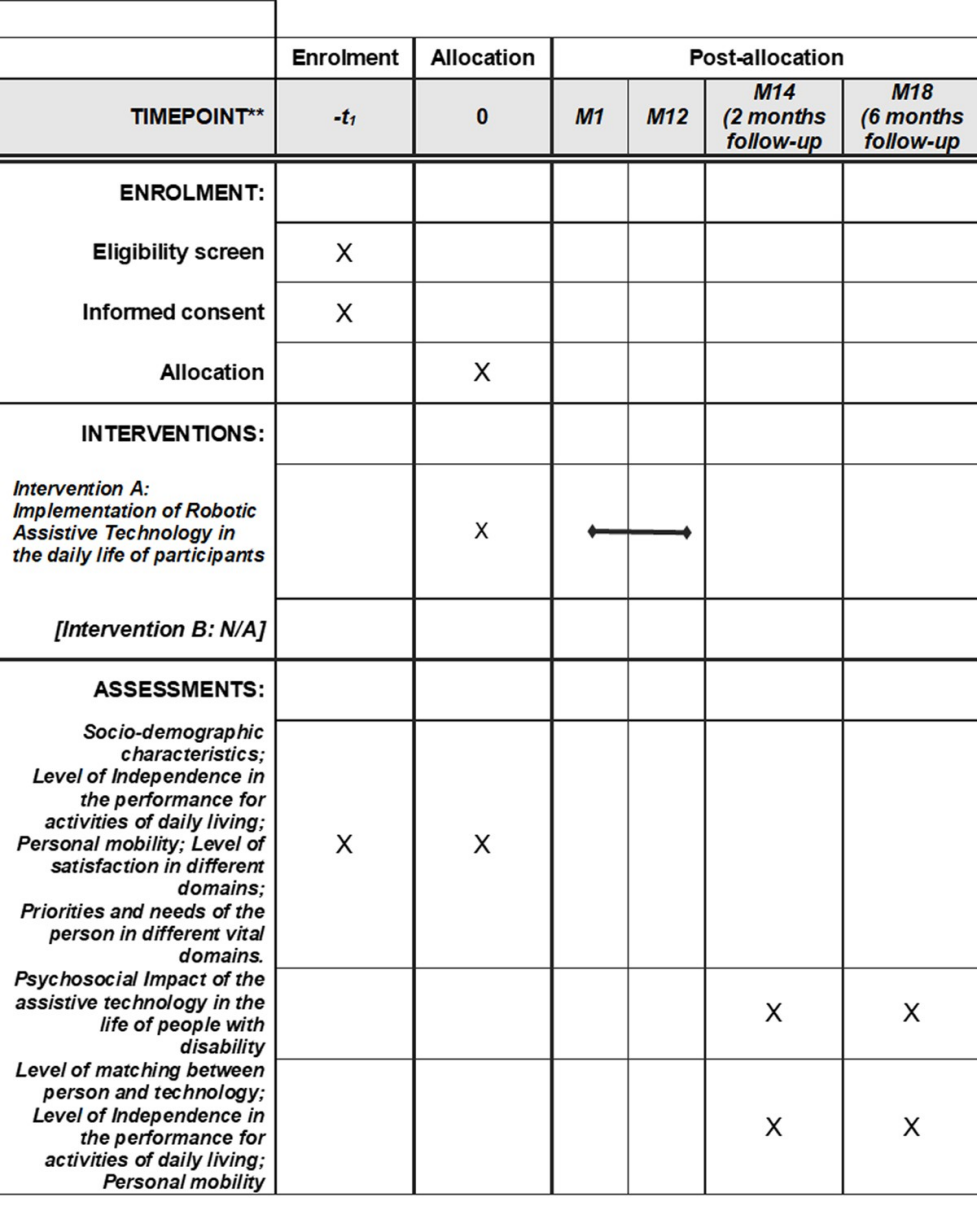
Schedule of enrolment, interventions, and assessments.

Fig 1 shows the schedule of the intervention and assessment included in this research, according to the Standard Protocol Items: Recommendations for Interventional Trials (SPIRIT Statement).

#### Description of assistive technology based on robotics and AI

The AT solution that is being implemented in the project is a low-cost mobile robotic platform that combines advanced navigation and visual perception capabilities, based on state-of-the-art Artificial Intelligence (AI) models. In addition, it incorporates a wearable device-based interface that records both biosignals and movement measurements of the subject. It achieves this by using sensors placed in different parts of the body that capture the physiological and inertial signals.

The main objective of the platform is to assist people with disability, monitoring some of their daily life actions in a controlled environment, using the robot and the wearable interface. We intend that the platform allows them to improve their independence and reinforce the learning of these activities.

**Fig 2:**
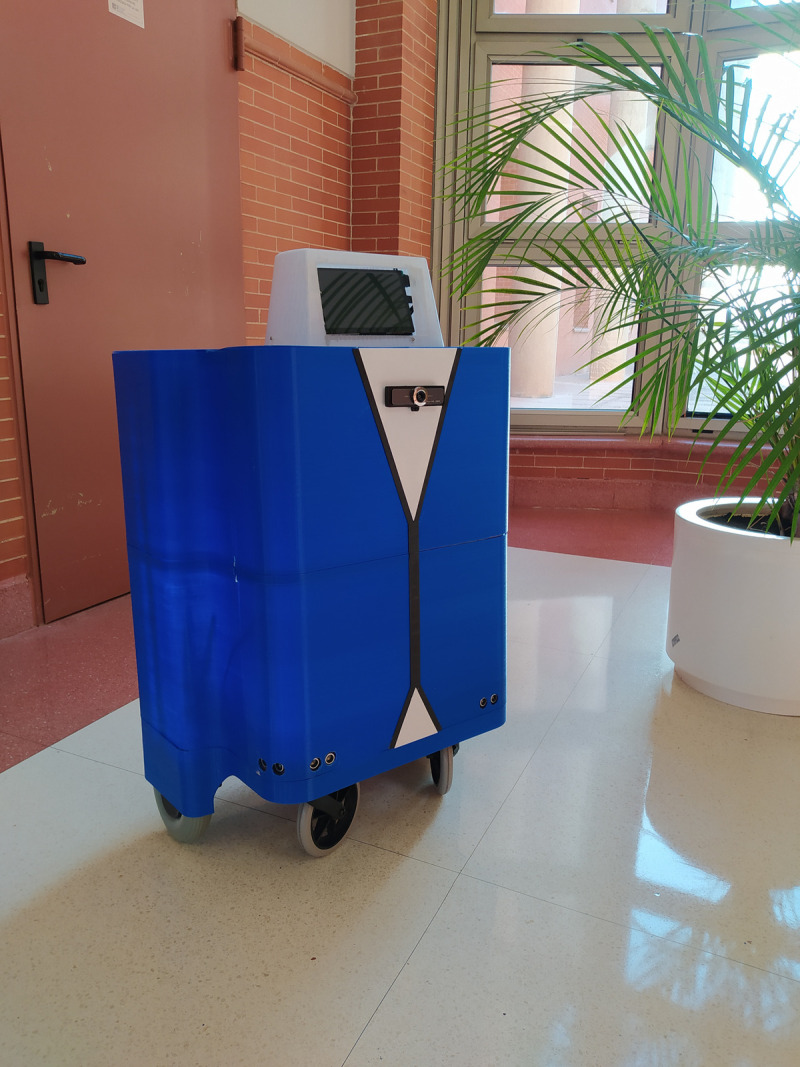
Prototype of AT.

Technically, the robotic platform (see Fig 2), named LOLA [20], has been entirely designed and constructed by the UAH team in the project. It is a differential wheeled robot, equipped with two motors and their corresponding encoders, which are controlled with an Arduino board. The internal structure is constructed of wood and metal. The outer shell, imitating a person wearing a tuxedo, was made entirely by 3D printing. The complete platform measures approximately 800 mm, slightly higher than a table. As for the sensors, the platform has: one LIDAR, a touch screen and a frontal camera. The LIDAR takes the measurements of the obstacles used for navigation and localization purposes. Finally, LOLA includes a Jetson Xavier board that has ROS integrated. It is in this board where all the AI functionalities are embedded.

For this study, the platform will navigate autonomously in certain controlled environments, defined by the centers with which we collaborate in the project. The intervention will mainly consist of reinforcing the learning of some activities of daily living. For this purpose, LOLA has integrated the following artificial intelligence algorithms:

1. Advanced semantic visual navigation solutions, that allow the robot to autonomously navigate through the environment, interacting with the users. Our solution improves the generalization capabilities of traditional Deep RL visual navigation approaches, which normally are prone to overfitting to their training environments. Therefore, the platform includes a visual semantic navigation module that can learn how to navigate in novel environments, without too much supervision. This is accomplished by integrating meta-learning procedures into the navigation module.
2. Novel online Action Detection (OAD) approaches allow the robot to monitor the actions performed by the users. This monitoring is performed in real-time. In this way, the developed software can generate an accurate report of when and with what degree of performance, the user has performed the activities. Technically, LOLA integrates a module for OAD which is based on the use of 3D Convolutional Networks (CNNs).

In addition to employing the LOLA platform, a set of smart wearable sensors, developed by the US team, will be used to monitor users. A better human-robot experience will be based on the integration of local wearable sensors to collect the subject’s physiological data for both emotional state detection and activity recognition. Physiological data mainly serve for monitoring the workout and the emotional state, while video processing allows determining the level of intensity and quality of the exercise.

This way, the mobile intelligent platform can autonomously navigate in a room to approach a person with a disability for helping him/her to develop a particular activity. At the same time, wearable technology has been monitoring the activity of the person and the mobile platform can enrich this monitoring by using the camera sensors embedded in it. At this point, both technologies can interact to facilitate the assessment by health personnel, or even to produce a more complete report on the performance of the participant’s activity. The use case proposed in this research involves reinforcing the learning of a set of activities of daily living. Specifically, the researchers have defined the following activities and their tasks to be used in the intervention with the platform: brushing teeth, cutting in the kitchen, handstand pushups, typing, writing on board, combing, eating and drinking. The LOLA platform has a screen (see the prototype in Fig 2) on which it will first show the users videos of the daily life activity that they have to reinforce. Once the video has been watched, LOLA will prompt the user to repeat the action, and then the monitoring system based on AI, computer vision and wearable devices will start up. During the development of the activity, the system will automatically generate a detailed report, which will include the following measurements (among others): time of development of the actions, whether they were performed correctly (online action recognition), recording of the user’s joints poses, physiological variables recorded by the wearable devices.

The interaction of the mobile platform with the intelligent wearable monitoring technology, and the adequate assessment to measure the impact on disabled people allow going further in terms of application possibilities for improving functional mobility and the independence in the performance of activities for people with disability.

#### Procedure

The main tasks of our research will be developed according to the following schedule:

1. Planning of assessment process: The research group defines the study variables and measurement instruments to obtain the data. These tools will allow determining the needs, capabilities, priorities and expectations of participants, concerning the use of AT.
2. Contact with the collaboration centers and presentation of the project: The members of each research group will contact the collaboration centers to present the characteristics of the project, to get their authorizations and, to acquire all the information about the potential participants.
3. Selection of participants and process of informed consent: According to the inclusion criteria, the participants will be recruited from each center, and the information sheet will be given to them. After reading the document, participants must provide their informed consent to be involved in the research.
4. Initial assessment: In this phase, the evaluation of the participants will be done, with the application of the instruments presented in Table 1. According to the age of the person, specific questionnaires will be used to gather more concrete information about the skills for mobility and functional independence. This assessment will allow determining the specific needs, demands and capabilities of participants. The assessment process will be done by professionals of the research groups directly in the collaboration centers. Depending on the instrument, and of the participant level for understanding the instrument questions, the assessment could implicate the parents (or legal guardians) of the children
5. Technical advice to the development of the robotic AT developed: According to the obtained results from the initial assessment and after the analysis of the first data, the research group of UDC will inform the groups of UAH and US of the main findings. The initial assessment allows knowing the participant’s needs, skills, and mobility capacity, considering the requirements that will be taken into account and incorporated into the technical development of the AT. The prototype of AT (see Fig 2) is a novel own design. The use of intelligent techniques integrated on the AT, will facilitate the fact of this solution can adapt itself in order to answer the specific needs of each participant. Moreover, the technical advice implies the advice from the research group of UDC about the specification of wearables used (design and type of body restraint) and the type of activities (task, movements and videos) that the mobile platform will show the user, on its screen, during the intervention. Only in this way, we will generate viable and adequate products for the previously detected needs, and whether this assistive technology, which integrates the robotic and AI models, answers correctly to these identified demands. With this in mind, is possible to create the AT with the approach of user-centered design.
6. Check of functioning and adaptation of assistive technology to the requirements of people with disabilities: The research group of UDC will verify the robotic AT in terms of usability and universal design, and its adequacy to the specifications identified in the previous phase. That process will be a collaborative task, in which the members of the research group and the professionals of collaboration centers will participate, without needing a direct intervention with the users.
7. Implementation of AT in the daily life of participants: The professionals of collaboration centers, supported by the research groups, will carry out the training in the use of AT by the participants, facilitating its incorporation during the performance of activities of daily living. Each participant will have a personalized program for intervention, with his/her own goals to improve functional mobility and independence. The activities will carry on in the environment of the collaborator center, and according to the goals established for each user. The time for the sessions using the AT solution will be 2 hours per week, divided into two sessions weekly. This phase will last 12 months.
8. Determination of possible improvements of the robotic AT, derived by the experience of participants: The members of the research groups will test the AT in the context of people with disabilities, detecting and registering the possible lacks and improvements needed to get the best match between the AT and the user.
9. Application of outcome measures and data analysis: After the process of implementation of AT (the next 2 and 6 months), the members of research groups and the professionals of collaboration centers will apply the measurement instruments to determine the possible improvements in the level of functional independence (FIM scale) of people with disabilities, the impact that the AT has had on their lives (PIADS scale) and the level of matching between the person and the AT (ATD PA–MPT model). The data will be analyzed in descriptive and inferential terms, establishing the respective comparison with the obtained results of the initial assessment.
10. Elaboration of an explicative model: The obtained results from the use of AT by the participants, and its posterior analysis will allow establishing the influencing factors that could facilitate or restrict the good and efficient use of this device. That will be the best solution to demonstrate the efficacy and efficiency of our work and its final goal to improve the participation and quality of life of people with disabilities.

**Fig 3:**
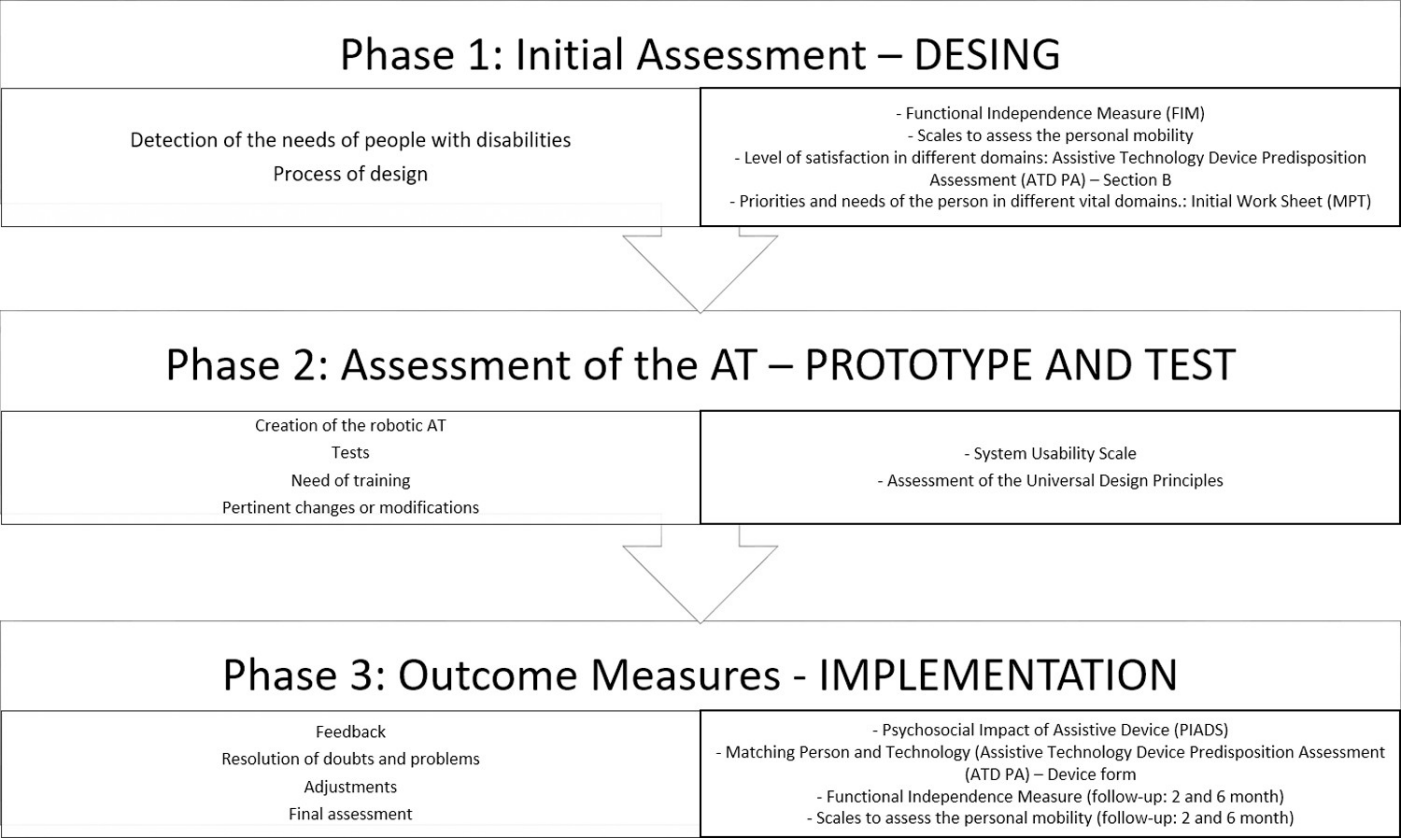
Structure of the assessment process.

In Fig 3, the structure of the assessment process and the use of outcome measures is presented.

The process of designing, developing and integrating the different features of the AT solution lasts 14 months. At the same time, the actions of selecting participants and starting the initial assessment are done. The research team has formed by (1) health and social professionals (occupational therapist, physical therapist, nurses and educators of special schools) who are in charge of selecting and evaluating the participants, as well as carrying out the intervention with the TA; and by (2) informatics engineer, physicist, and electronic engineer, that develop the technological solution, integrating these components and do the lab tests. Periodic meetings are needed in order to incorporate the technical advice, based on the results from the evaluation, and to design the AT solution taking into account the real needs and expectations from participants.

### Ethical considerations

All participants will receive a piece of research-related information about the project.

The informed consent sheet has been written in a language that can be easily understood by the subjects, and it will be obtained from all participants or parents/legal tutors when involving children. There is no obligation to complete the experiment so that participants can withdraw from it at any time. The participation of the users and the centers of intervention is totally free for them. The treatment of biological samples or research with drugs or medical devices is not contemplated.

The performance of this study will respect the applicable regulations of ethics regard to research with humans. Mainly, the following legislative documents will be applied: Declaration of Helsinki: Ethical principles for medical research involving human subjects (version 9th July 2018); European Commission. Ethics for researchers: Brussels: European Commission (2013); The Oviedo Convention: Protecting human rights in the biomedical field (4th April 1997); Law 14/2007, of biomedical research; and Law 3/2005, of informed consent and clinical history of patients. For data protection, we will follow “Regulation (EU) 2016/679 of the European Parliament and of the Council of 27 April 2016” and the Spanish law: “Ley Orgánica 3/2018, del 5 de diciembre, de Protección de Datos Personales y garantía de los derechos digitales”.

The confidentiality of the data and the anonymity of the data will be maintained through pseudonymization techniques. Once the study is finished, the data will be stored anonymously, with prior authorization from the participants.

The platform used for the registration and analysis of the results will be RedCAP (Research Electronic Data CAPture). “*That is a secure web application for building and managing online surveys and databases*. *While RedCAP can be used to collect virtually any type of data in any environment (including compliance with 21 CFR Part 11*, *FISMA*, *HIPAA*, *and GDPR)*, *it is specifically geared to support online and offline data capture for research studies and operations*.” The use of RedCAP will allow to codify previously the data, and guarded them with specific passwords of very high privacy [19].

This research has been approved by the Galician Research Ethics Committee, with the reference number: 2020/597, in February, 2021. Also, it is registered in ClinicalTrials register with the ID: NCT04723784

### Data analysis

The quantitative variables will be expressed as Mean (M) and Standard Deviation (SD), including range, minimum and maximum values. Apart from the simple description of data and variables, the inferential analysis will be done to determine possible significant relationships and correlations between study variables and that allows the contrast of hypothesis.

Shapiro Wilk and/or Kolmorogov–Smirnov will be applied to determine if the sample meets the normal distribution criteria, and to decide the type of statistical test that will be implemented (parametric or non-parametric). The T- student paired test or the U-Mann Whitney will be applied to establish the possible differences of means, according to different dichotomic variables, in the assessment scales of FIM, PIADS, Walk tests and level of matching person–AT. The ANOVA test or Kruskal–Wallis will be used in the cases of polychotomic variables, to establish possible differences of means between results of the scales. The association of numeric variables will be analyzed by Pearson or Spearman correlation tests, according to the distribution of the sample. To evaluate the association of categorical variables, chi-square will be applied, or likelihood ratio if the observed frequencies are lower than 5%. To check the differences between related samples, with repeated measures (in the case of FIM scale and the scales of mobility–initial assessment and in the follow-up of 2 and 6 months), the t-student test or the Wilcoxon ranges will be used, according to correspond to the sample distribution.

The analysis of the data will be done with the program SPSS v.24® for Windows. The level of significance to do the contrast of hypothesis is set to 5%.

## Discussion

The development of this research aims to go beyond the state of the art in new directions, such as the creation of a low-cost assistive robotic platform to improve the independence and QoL of people with disabilities, and the possibility of detecting emotional states in people with severe communication difficulties.

It is noted that people with disabilities have a lot of barriers to access and to do the activities that they want to do, most of the time conditioned by environmental factors and the lack of the correct support [4,21,22]. Assistive Technology is a possible solution to increase the participation and performance of this group, but actually, the available devices in the market don’t meet completely their needs, neither they take into account the individuality of each person with a disability [23–25]. With the development of the AIR4DP project, the match between person and technology will be increased. The AT solution of this project incorporates features of AI, that allow adapting its responses for the needs and demands of each participant. If it meets the person’s performance expectations and is easy and comfortable to use, then a good match of person and technology has been achieved [5]. This goal will be succeeded with the incorporation of the structure of the assessment process and the application of outcome measures.

### Socio-economic impact

The development of new healthcare techniques will improve the quality of life of people with different types of disabilities. Likewise, the sometimes prohibitive price of many of the solutions on the market, imposes on the researchers, the task of making this type of system more accessible to the general public [14,26]. We include in this group, both people with disabilities as individuals, as well as schools, medical centers, or institutions that perform this social initiative. Moreover, the other significant problem is that if the AT is not adapted to individual needs or doesn’t fit his/her expectations, it is not used [23].

The field of disability is a niche in which research, design, and innovation can be applied to a social purpose.

The perception of the uses is that it is necessary to apply technology to the field of disability and, this application must be achieved at reasonable costs. We understand that a proper connection between research, development, and the needs of disabled people can be an opportunity to create wealth and knowledge.

### Transfer plan

The consortium is well aware of the great importance of transfer efforts in the context of attention to disability. Without the transfer, there is no help. That is why with this work we intend to bring aid to interested centers and patients. For doing so, the research groups have contacted some institutions, which are interested in the outcomes of the project, and most of them will participate actively in the development of the research.

Another of the transfer tasks that we will explore, will consist in the elaboration and presentation of patents. We hope to be able to materialize some of the most relevant scientific findings in one or several patents, which can then be exploited by some companies interested in the sector of assistive technology and healthcare.

The coordinated project has received funding from the Ministry of Science, Innovation and Universities (Government of Spain). The main expected result of this project is the implementation of assistive technology (AT) that allows incorporating the last innovations in Artificial Intelligence (AI) and robotics to improve the Quality of Life (QoL) of people with disabilities.

## Supporting information

S1 Checklist Spirit statement checklist. (PDF)

S1 File Ethics committee approval letter and translation to English. (PDF)
