# Supplementary material for: Assessment and counseling to get the best efficiency and effectiveness of the assistive technology (MATCH): Study protocol
Source: PLoS One. 2022 Mar 16;17(3):e0265466. doi: 10.1371/journal.pone.0265466 (PMC8926266; doi:10.1371/journal.pone.0265466)
Supplement: S1 File — (PDF) [file pone.0265466.s002.pdf]

TITLE: Assess**M**ent and counseling to get the best efficiency and effectiveness of the **A**ssistive **T**e**CH**nology **(MATCH)**

NCT number: NCT04723784

Date of document: 20/01/2021

Institutions:
University of A Coruña
University of Alcalá de Henares
University of Sevilla

Abstract:

The best match between any person with disability and the assistive technology (AT) only can be gotten through a complete assessment and a monitoring of the needs, abilities, priorities, difficult and limitations that he/she finds in his/her life. Without this analysis, may be the risk that the AT doesn't adapt to the expectation of the person. Like this, the user, in few time, could abandon the AT device, with the unnecessary spends of resources. Therefore, is important that any project focused in the development of new innovating AT for people with disabilities includes the perspective of outcome measures as an important phase of the research. In this sense, the incorporation of the assessment, implementation process and outcome measures is vital to get the transferability during the whole project and to get the general perspective from the final user.

The protocol presents a project that aims to improve the independence, participation and functional mobility of people with disabilities.

The sample is formed by people with disabilities that will participate from the first stage of the process, with an initial assessment of their abilities and needs, a complete implication during the test of technology, and in the final application of outcome measures instruments.

Only with this perspective and active participation of the users is possible to carry on a user-centered approach. That fact will allow to define and to generate technological solutions that really adjust to the expectations, needs and priorities of the people with disabilities, avoiding that the AT be abandoned, with the consequent health and social spending.

1. **Introduction:**

The present document is the description of a Subproject of a global and complete coordinated Project, in which are participants the University of A Coruña, the University of Alcalá de Henares and the University of Sevilla, and its tittle is: Artificial Intelligence and Robotic Assistive Technology devices for Disabled People (AIR4DP).

That coordinated project has the funding by the Ministry of Science, Innovation and Universities (government of Spain), through the call "Proyectos I+D+i 2019 – Retos de la Sociedad)", reference: PID2019-104323RB. The main expected result of this project is the implementation of assistive technology (AT) that allows to incorporate the last innovations in artificial intelligence and mobility to improve the quality of life (QoL) of people with disabilities.

The three subprojects that form part of the coordinated one are:

- Artificial Intelligence and Robotic Mobile Platforms to Improve Disabled People Independence (AIRPLANE): AIRPLANE is presented by the University of Alcalá (UAH). It aims to offer to the AIR4DP project advanced mobile technology solutions to improve the independence of people with functional diversity. The platforms are able to autonomously navigate the environment, e.g., the house of the user with functional diversity, and track the activity of the people, anticipating dangerous situations, such as a fall or an awkward interaction with a household appliance.
- Augmentative Affective Interface (AAI): Searches for a better human-robot experience by including remote monitoring and local wearable sensors to collect the subject's physiological data for both emotional state detection and activity recognition. Namely, AAI looks for improving the quality of life of people with disabilities by adapting and promoting physical activity autonomously but in a controlled scenario.
- Assessment and counseling to get the best efficiency and effectiveness of the Assistive Technology (MATCH): That subproject is the complement to get the best evidence about the impact of assistive technology based on AI in the life of a person with a disability. The perfect match between person and technology can only be achieved through a complete assessment and monitor of the capabilities, needs, priorities, difficulties, and limitations that a person has in their life. Without these analyses, there is a risk that the technology does not adapt to the expectations of the person. The person in a short time abandons the project with the result of unnecessary expenditure of resources. So, it is relevant that within the coordinated project, a team with experience in evaluation, design, and outcome measures of assistive technology for people with disabilities is integrated in the consortium.

The main result of the AIR4DP project consists in the implementation of assistive technology that allows us to incorporate the latest advances in AI to improve life quality of people with disabilities. With this target, we present a coordinated project that is divided into three sub-projects, whose main goals consist in developing and evaluating the breakthrough of the proposed technology in the people with functional diversity. In a nutshell, AIR4DP main scientific objectives are:

1. Create novel mobile platforms with advanced perception, interaction and navigation capabilities, able to stimulate and monitor the improvement of the independence of people with functional diversity (SUBPROJECT 1: AIRPLANE).

2. Develop new wearable technical solutions for monitoring physiological aspects to help the detection of emotional states and user activities (SUBPROJECT 2: AAI).
3. Get the best evidence about the impact of assistive technology based on AI in the life of the person with a disability (SUBPROJECT 3: MATCH).

The consortium formed allows us to cover from the implementation of intelligent mobile platforms to improve independence (UAH) and multi-modal wearable sensor solutions for monitoring people (US), to the assessment, from a health point of view, of the impact of these aids on people with functional diversity (UDC, UAH).
In this Study Protocol the justification and the development of subproject MATCH is explained. It will be coordinated by the University of A Coruña, and its performance implies the participation of people with disabilities.

2. Background:

Assistive Technology (AT) is an umbrella's concept that refers to devices with different levels of technological complexity. AT improve the functional capacity in different areas of a person's life. Some of those areas are: a) eating (for example, adapted cutlery, dishes with flange); b) mobility (for example, powered wheelchairs, walkers, canes); c) communication (for example, augmentative and alternative communication systems); d) access to the computer (for example, screen readers, adaptations for keyboards, push buttons, computer systems, switch); e) housing; f) transport; and g) leisure or sports (1). Therefore, the AT are devices that facilitate and/or allow the development of activities of a person with a disability.

However, despite the clear benefits of the use of assistive technology devices (ATD) in the life of people with disabilities, it has also been associated with some problems. The more common ones can be grouped into several categories: lack of information about available ATD, economic factors related to the high price of products, forms of access to support products, as well as factors related to the guarantee, maintenance and insurance of the ATD (2–4).

One of the most frequent and influential factors during the process of prescription and subsequent use of the ATD, is the lack of matching between the person and the recommended ATD (5,6)**.** This problem may lead to the abandonment or non-use of the ATD. The non-use of devices has clear causes and obvious consequences not only on the quality of life and the autonomy of the person with disability but also cost-benefit effects (7). To achieve an increase in the successful use of ATD and, therefore, to reduce the probability of being abandoned or not fulfilling its initial function, it is essential to carry out an adequate assessment of the user's needs with reliable measurement tolls to improve decision making for the prescription and adaptations of ATD.

The outcome measure is a set of considerations and tools to determine if a service, product or device allows achieving the goals for which it has been created, under criteria of efficacy, and effectiveness. In other words, outcome measures are the evaluation process in the provisioning service that is designed to quantify and establish a baseline on something that works (its effectiveness), the group on which it works, and what level of economic efficiency it provides (8). So, professionals prescribing AT have to take into account the need for applying outcome measures to improve their intervention, with a process evidence-based.

WHO General Assembly in 2018 published a resolution on the importance of ATD and services globally (including assessment) and supported the position papers. The main considerations that

have to take into account the governments in the provision of assistive technology to all citizens are related to Personal, Products, Provision, and Policy (9). To give support to the professionals, to improve the services of AT and to benefit the final users of those devices, several models of outcome measures have emerged (6,10). Those models indicated the main factors to take into account in the process of getting a match between the person and assistive technology:

- The functional problems that ATD intends to solve.
- The characteristics of final users and their needs and priorities.
- The characteristics of the device that are responsible for its intervention.
- The context in which ATD is applied or used.
- The expected changes in the state of a user and its context are the results, both short and long term.
- The impact of AT devices on the individual's participation in the environment.

A revision done by the ATOMs Project identified 22 models of outcome measures related to ATD, and 14 tools or instruments to assess the results of that device, such as, psychosocial impact of ATD in the quality of life, satisfaction with the ATD or level of matching between person and technology (11). In Spain, the research related to outcome measures in ATD is deficient, because there are not many specific measurement instruments that are validated in the Spanish population. The application of these tools was published only in two research projects (12,13). The PhD thesis of the principal investigator (Thais Pousada) of the MATCH subproject and all the research after that is the only available expertise in that topic (7,14–16).

Therefore, the third subproject of this proposal is focused on the implementation of outcome measurement tools to improve the efficacy, effectiveness and the real utility of the ATD developed. On the other hand, it is also very important to assess the functional impact the ATD has on user's participation in their social environment. For that, other measurement tools to assess functional mobility outcomes will also be also carried out during basal assessment and after 6 months of ATD implementation. The mentioned tools include (all in their Spanish adapted version) the Six-minute Walk Test (17,18), 10 Meter Walk Test (19), Pediatric Balance Scale (20), and the Functional Independence Measure (FIM) (21,22). The measurement tools will be carried out depending on the characteristics of the ATD and the targeted population.

3. Objectives and initial hypothesis

The global project is a multi-disciplinary and cross-border research, where the **main research objective consists in the development of novel AI and computer vision based robotic solutions with advanced mobility and human-robot wearable interaction capabilities, to improve the independence and stimulation of people with disabilities**.

The general objective of this project is based on the hypothesis that the application of Information and Communication Technologies (ICTs) and AI can improve the quality of life and independence in the group of people with disability by providing support platforms to enhance the participation in activities of daily life and in their communities.
Overall, with the coordinated research we will address the following open questions:

- What techniques can be proposed from the perspective of artificial intelligence and computer vision to provide effective solutions for people with functional diversity?
- What are the challenges posed by human-robot interaction, using accompaniment platforms and wearable devices, with people with disabilities?
- How should we approach evaluation and consultancy for the design of robotic solutions as assistive technologies, so that we can guarantee an efficient and correct matching between person and technology?

In concrete, for this study protocol, the second hypothesis is related to the relevance of the assessment of the interaction between the users and the AT: in order to guarantee a high match person-AT, to also get a positive psychosocial impact in the life of people with disabilities, the assessment and monitoring result two fundamental aspects.

According to this hypothesis, the research groups has fixed two main objectives for the MATCH subproject:

- To determine the psychosocial impact of AT created during the coordinated project (AIR4ALL) on the life of people with disabilities.
- To promote the best match between the user and the assistive technology used.

To complete, few secondary goals have been proposed:

- To assess the functional skills and abilities of people with disabilities, and to identify their needs to get independence in mobility and activities of daily living.
- To design and create the best technology solutions individualized for each person, meeting with the design for all principles, and promoting the involvement of the final user.
- To validate the assistive technology's solutions designed and prototyped in order to increase the levels of participation in daily activities to people with disabilities.
- To validate a protocol/model of outcome measures in the field of assistive technology to increase the efficiency and effectively of selecting and prescribing these products.

4. Methodology

Design: Pilot study: Prospective, longitudinal and analytical cohort

Environment of study:

The project will be done by researchers of Universities of Alcalá de Henares, Sevilla and A Coruña. So, the context of study is restricted to that locations.

In the case of University of Alcalá de Henares, the context is located to this city and few districts of Madrid. The centers of people with disabilities that will be able to participate are: Centro de Atención Integral SAIDI-APHISA, Fundación ASTOR, Colegio Público de Educación Infantil y Primaria (CEIP) Luis Vives, Colegio Público de Educación Especial Pablo Picasso and ATENPACE.

The University of Sevilla will carry on the project in the city of Sevilla and its metropolitan area. The collaborators centers will be: ASPACE Sevilla and Colegio de Educación Especial Directora Mercedes Sanromá.

Finally, in the case of University of A Coruña, its context of study includes the metropolitan area of A Coruña and Santiago de Compostela, conditioned by the influence area of its collaborators: ASPACE Coruña and Confederación Gallega de Personas con Discapacidad (COGAMI).

Period of study:

The coordinated project has a duration of 36 months, and it has been programmed from the month of July 2020 to the month of April of 2023.

Sample and selection of participants:

The participants will be those that meet with the inclusion criteria and not the exclusion ones, and they will have given their informed consent to participate in the research.

The inclusion criteria are the following:

- People with recognized disability, derived by a disease or a permanent health status.
- Age between 2 and 21 years old.
- To form part of any of collaborators centers with the three universities.
- To have an independent functional level moderate – low (assessed by the Functional Independence Measure.
- To have functional skills – mobility domain level moderate – low (assessed by the Spanish version of Pediatric of Disability Inventory – PEDI)

The exlusion criteria are:

- To have any health status that is incompatible with the use of assistive technology designed and prototyped in the project.
- To have cognitive skills very limited, that limits to follow the instructions to good use of AT.
- Not to have adequate human supports to make use of AT.
- To have functional skills – mobility domain level low (assessed by the Spanish version of Pediatric of Disability Inventory – PEDI), with the need for maximum assistance from caregivers.

The research group have also fixed *criteria for retirement* of study's participants:

- Voluntary abandonment of the research and/or to finish the relationship with the center where the research project is carrying on.

Selection of participants:

All persons that meet with inclusion criteria will be invited to participate in the research. The selection of participants will be done by each research group (UAH, US and UDC), through their contact with collaborators centers (specified below). In all cases, an information letter with the whole information of the project will be done to the chief or directors of those centers, to get the authorization to implement the research project in that entity.

Variables and Instruments:

The study variables and the measure instruments correspondent are specified in Table 1.

The use and the application of each instrument is conditioned by the stage of intervention process, that is defined and described in the next section. All instruments have been translated and validated into the Spanish context.

The access to the clinic history of the participants won't be required to the performance of this project.

Nevertheless, all obtained results will be codified and manage through the REDAP Platform, that allows the capture of electronic data in the design of research database

Procedure:

The performance of MATCH subproject will be conditioned by the advance of other two subprojects. Nevertheless, in the schedule (see below) is showed how the different tasks are related to each other, and how the development of the complete project will be in the timeline.

The tasks that will be done in the MATCH subproject contemplates several consecutive phases:

1. Planning of assessment process: The research group defines the study variables and measure instruments to obtain the data. Those tools will allow to determine the needs, capabilities, priorities and expectations of participants, with respect to the use of AT.
2. Contact with the collaborators centers and presentation of the project: The members of each research group will contact with the collaborators centers (specified in sections before) to present the characteristics of the project, to get its authorization and, at the time, to give all information of the users, potential participants.
3. Selection of participants and process of informed consent: According to the inclusion criteria, the participants will be recruitment of each collaborator center, and the information form will be given to them. After the reading, participants will offer their informed consent to involve in the research.
4. Initial assessment: In this phase the evaluation of the participants will be done, with the application of the instrument presented in table 1. According to the age of the person, specific questionnaires will be used to gather information more concrete about the skills for mobility. This assessment will allow determining the specific needs, demands and capabilities of participants. The assessment process will be done by professionals of research group in rooms of collaborators centers.
5. Technical advice to the development of the other subprojects (AIRPLANE and AAI): According to the obtained results from initial assessment and after the analysis of first data, the research group of UDC will inform to the groups of UAH and US the main findings. That data will be taken into account and incorporated to the technical development of the AT. Only in this way, it will generate viable and adequate products to the detected needs previously, and this assistive technology that integrates the robotic and AI models answer correctly to these demands.
6. Check of functioning and adaptation of assistive technology to the requirements of people with disabilities: The research group of UDC will verify the components of assistive technology in terms of usability and universal design, and its adequacy to the specifications identified in the previous phase. That process will be a collaborative task, in which will participate the members of research group and the professionals of collaborator centers, not implicating a direct intervention with the users.

7. Implementation of AT in the daily life of participants: The professionals of collaborator centers, supported by the research groups, will carry on the training in the use of AT by the participants, facilitating its incorporation during the performance of activities of daily living.
8. Determination of possible improvements in the assistive technology, derived by the experience of participants: The members of research group will test the AT in the context of people with disabilities, detecting and registering the possible lacks and improvements that the device would need to get the best match between the AT and the user.
9. Application of outcome measures and analysis of the data: After the process of implementation of AT (the next 2 and 6 months), members of research group and the professionals of collaborators centers will apply the measure instruments to determine the possible improvements in the level of functional independence (FIM scale) of people with disabilities, the impact that the AT has had on their lifes (PIADS scale) and the level of matching between person and AT (ATD PA – MPT model). The data will be analysed in descriptive and inferential terms, establishing the respective comparison with the obtained results of initial assessment.
10. Elaboration of explicative model or protocol: The obtained results from the use of AT by the participants, and its posterior analysis will allow to determine the influent factors that would be able to facilitate or restrict the good and efficient use of this device. That will be the best solution to demonstrate the efficacy and efficiency of AIR4ALL and its final goal to improve the participation and quality of life of people with disabilities.

Data analysis:

The quantitative variables will be expressed as Mean (M) and Standard Deviation (SD), including range, minimum and maximum values. Apart from the simple description of data and variables, inferential analysis will be done to determine possible significant relationships and correlations between study variables or that allows to the contrast of hypothesis.

Shapiro Wilk and/or Kolmorogov – Smirnov will be applied to determine if the sample meets with the normal distribution criteria, and to decide the type of statistical test that will be implemented (parametric or no-parametric).

The T- student paired test or the U-Mann Whitney will be applied to stablish the possible differences of means, according different dicotomic variables, in the assessment scales of FIM, PIADS, Walk tests and level of matching person – AT. The ANOVA test or Kruskal – Wallis will be used in the cases of polycotomic variables, to stablish possible differences of means between results of the scales.

The association of numeric variables will be analysed by Pearson or Spearman correlation test, according to distribution of the sample. To evaluate the association of categorical variables, chi-square will be applied, or likelihood ratio if the observed frequencies are lower than 5%.

To check the differences between related samples, with repeated measures (in the case of FIM scale and the scales of mobility – initial assessment and in the follow-up of 2 and 6 months), the t-student test or the Wilcoxon ranges will be used, according correspond to the sample distribution.

The analysis of the data will be done with the program SPSS v.24 for Windows. The level of significance to do the contrast of hypothesis is stablished to 5%.

5. Security and adverse effects

The damages or inconveniences that can derived from the participation in this research are related with the time dedicated to the application of the questionnaires or scales (assessment phases), and during the test of AT.

During the development of the project, in concrete in the phase of elaboration and training of AT, all measures of security and personal protection required will be taken into account, to avoid possible damage derived from the use of materials and products. Moreover, research group will write a guideline of basic norms, cautions and recommendations that help in the use of AT by professionals and participants.

In the information that will be given to the participants (information sheet), these inconveniences will be noted, to offering the possibility to retire in any moment, if they detect any adverse effect during the development of the research.

6. Planning and Schedule:

Figure 1 offers a synthesis of a development of MATCH subproject, indicating the work packages derived from the specified tasks indicated in the procedure.

- T1: Protocol of assessment and intervention to implement. Definition and concretion of the process of informed consent.
- T2: Report of results from the initial assessment.
- T3: Report of improvements of the prototype to the access and use of assistive technology.
- T4: Registration of the implementation sessions with the AT.
- T5: Report from the detected improvements after the process of implementation of AT.
- T6: Report from the statistical analysis.
- T7: Descriptive protocol of applying of outcome measures in developments of artificial intelligence and robotics on AT.

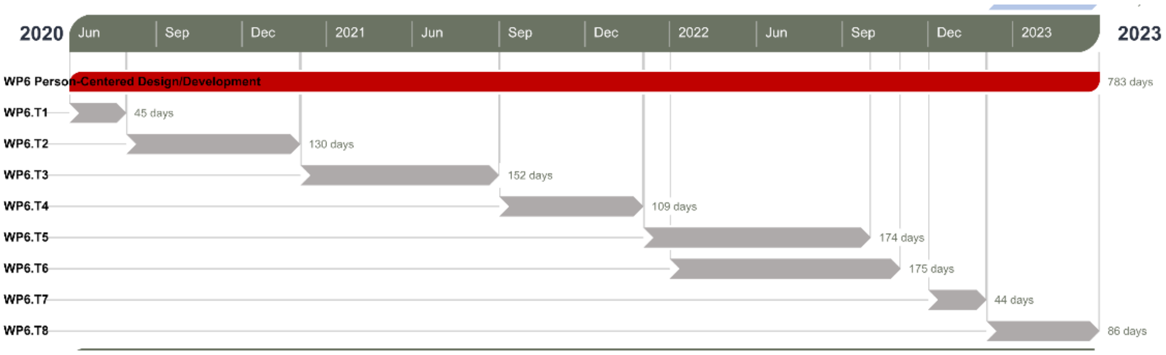

Figure 1: Schedule of development of MATCH subproject.

7. Ethical Considerations, Protocols and Procedures:

All participants will receive a piece of research-related information about the project.

The informed consent form must be written in a language easily understood by the subjects, and it will be obtained from all participants or parents/legal tutor when involving children. There is no obligation to complete the experiment so that participants can withdraw from it at any time.

The treatment of biological samples or research with drugs or medical devices is not contemplated.

The performance of this study will respect the applicable regulations of ethics regard to research with humans. Mainly, the following legislative documents will be applied:

- Declaration of Helsinki: Ethical principles for medical research involving human subjects: General Principles (version 9th July 2018);
- European Commission. Ethics for researchers: Facilitating research excellence in FP7. Brussels: European Commission (2013);
- The Oviedo Convention: Protecting human rights in the biomedical field (4th April 1997);
- Ley 14/2007, de 3 de julio, de Investigación biomédica;
- Ley 3/2005, del 7 de marzo, de modificación de la Ley 3/2001, del 28 de mayo de 2001, reguladora del consentimiento informado y de la historia clínica de los pacientes.

For data protection we will follow "Regulation (EU) 2016/679 of the European Parliament and of the Council of 27 April 2016" and "Ley Orgánica 3/2018, del 5 de diciembre, de Protección de Datos Personales y garantía de los derechos digitales".
The confidentiality of the data and the anonymity of the data will be maintained through pseudonymisation techniques. Once the study is finished, the data will be stored anonymously, with prior authorization from the participants.

The platform used to the registration and analysis of the results will be RedCAP (Research Electronic Data CAPture). "That is a secure web application for building and managing online surveys and databases. While REDCap can be used to collect virtually any type of data in any environment (including compliance with 21 CFR Part 11, FISMA, HIPAA, and GDPR), it is specifically geared to support online and offline data capture for research studies and operations." The use of ReCAP will allow to codify previously the data, and guarded with specific passwords of very high privacy.

This research has been approved by the Galician Research Ethics Committee, with the reference number: 2020/597.

8. Expected Results Impact:

- *Beyond state-of-the-art contributions*

The development of the project aims to go beyond the state of the art in new directions, such as the creation of low-cost assistive robotic platform to improve people's independence, and the possibility of detecting of emotional states in people with severe communication difficulties.
It is noted that people with disabilities have a lot of barriers to access and to do the activities that they want to do, most of the times conditioned by environmental factors and the lack of the correct support. The assistive technology is a possible solution to increase the participation and performance of this group, but actually, the available devices in the market don't meet

completely their needs, neither they take into account the individuality of each person with disability. With the development of AIR4DP, and with the contributions from all researchers' participants, especially in the application of outcome measures, the match between person and technology will be increased.

- *Socio-economic impact:*

The different analyzed reports on health and dependency show that the number of people with some disability, whether motor, sensorial, or cognitive, is sufficiently significant to justify the development of this project. Likewise, healthcare techniques and methods increasingly require ICT. Therefore, the development of new care techniques, in a relatively new area compared to other areas of research, will improve the quality of life of people with different types of disabilities. Likewise, the sometimes prohibitive price of many of the solutions on the market, imposes on the researchers, the task of making this type of system more accessible to the general public. We include in this group, both people with disabilities as individuals, as well as schools, medical centers, or institutions that perform this social initiative. Moreover, the other significant problem is that if AT is not adapted to individual needs or doesn't fit his/her expectations, it is not used.

The field of disability is a niche in which research, design, and innovation can be applied to a social purpose. In this sense, the document "Estrategia española sobre discapacidad. 2012-2020" of the Ministry of Health and Social Affairs, includes some figures and challenges that give an idea of the impact in this field. With regard to products and services linked to disability, this text said: "As for the existence of Information Society products or services accessible to DAPs, 82% agreed, although only 58% were able to name at least one accessible service or product, which demonstrates the gap between technologies and devices developed and their availability (at reasonable cost) in the market and therefore one of the objectives to be covered in this Strategy, even more so when 85% of those surveyed were optimistic that current or future Information Society technologies could solve the problems of DAPs". In this paragraph, we observe two of the parameters that are the starting point of the project presented. The users' perception that it is necessary to apply technology to the field of disability and, this application must be achieved at reasonable costs. In the conclusions of the document on disability strategies, it is mentioned in which areas and in which way the effort to implement solutions for disability will be expressed. It tells literally: "The identification of niches and market opportunities not yet covered or with scarce development should be promoted in this area, catalyzing new opportunities for research, development, and innovation, and fostering the connection between industry, business, and the university, as well as public-private partnerships". We understand that a proper connection between research, development, and the needs of disabled people can be an opportunity to create wealth and knowledge.

- *Transfer plan:*

The consortium is well aware of the great importance of transfer efforts in the context of attention to disability. Without transfer there is no help. That is why with this project we intend to bring aid to interested centers and patients. For doing so, research group have contacted with some institutions, which are interested in the outcomes of the project, and the most of them will participate actively in the performance of research project.

Another of the transfer tasks that we will explore, will consist in the elaboration and presentation of patents. As it can be seen in the trajectory of the research groups involved, they are all quite active in the granting of patents: UAH (4 patents), US (7 patents) and UDC (3 patents). We hope to be able to materialize some of the most relevant scientific findings in one or several patents, which can then be exploited by some companies interested in the sector of assistive technology.

References:

1. Groba B, Pousada T, Nieto L. Assistive technologies, tools and resources for the access and use of information and communication technologies by people with disabilities. Handbook of Research on Personal Autonomy Technologies and Disability Informatics. 2010.

2. Laloma M. Ayudas técnicas y discapacidad. Madrid: Comité Español de Representantes de Personas con Discapacidad; 2005.

3. VVAA. Proyecto EASTIN. Red europea de información sobre las tecnologías para la discapacidad y la autonomía. Minusval. 2005;153:17–34.

4. Vidal García Alonso J, Prat Pastor J, Rodríguez-Porreor Miret C, Sández Lacuesta J, Vera Luna P. Libro Blanco I+D+I al servicio de las Personas con Discapacidad y las Personas Mayores. Vol. 1. Madrid: Ministerio de trabajo y asuntos sociales, Ministerio de Ciencia y tecnología; 2003.

5. Scherer. Assistive technology matching device and consumer for successful rehabilitation. Washington DC: American psychological Association; 2001.

6. Scherer et al. A framework for modeling the selection of assistive technology devices (ATDs). Disab Rehab Assist Techn. 2007;2(1):1–8.

7. Pousada García T, Nieto Rivero L, Pereira Loureiro J, Díez Villoria E GGB, García TP, González BG, Rivero LN, Loureiro JP, Villoria ED, et al. Exploring the Psychosocial Impact of Wheelchair and Contextual Factors on Quality of Life of People with Neuromuscular Disorders. Assist Technol. 2015;27(4).

8. DeRuyter. The importance of outcome measures for assistive tehcnology service delivery systems. Technol Disabil. 1997;6:89–104.

9. World Health Organization. Global priority research agenda for improving access to high-quality affordable assistive technology The Initiative [Internet]. Geneva; 2017 [cited 2017 Jul 18]. Available from: http://apps.who.int/iris/bitstream/10665/254660/1/WHO-EMP-IAU-2017.02-eng.pdf?ua=1

10. Fuhrer, Jutai, Scherer, Ruyter D. A framework for the conceptual modeling of assistive technology device outcomes. Disabil Rehabil. 2003;25(22):1243–51.

11. Smith RO, Rust KL, Jansen C, & Seitz J. ATOMS Project technical report - The ICF in the context of assistive technology (AT) interventions and outcomes [Internet]. Vol. 2010. 2006. Available from: http://www.r2d2.uwm.edu/atoms/archive/icf.html.

12. Jimenez Arberas E. “Impacto psicosocial de los productos y tecnologías de apoyo para la

comunicación en personas con discapacidad auditiva y personas sordas [Internet]. University of Salamanca; 2016. Available from: http://sid.usal.es/idocs/F8/FDO27380/JimenezArberas.pdf

13. Pousada Garcia T. Impacto psicosocial de la silla de ruedas en la vida de las personas con enfermerades neuromusculares. [A Coruña]: Universidade da Coruña; 2011.

14. Groba B, Pousada T, Nieto L. Assistive technologies, tools and resources for the access and use of information and communication technologies by people with disabilities. Assistive Technologies: Concepts, Methodologies, Tools, and Applications. 2013.

15. Pousada T, Groba B, Nieto-Riveiro L, Pazos A, Díez E, Pereira J. Determining the burden of the family caregivers of people with neuromuscular diseases who use a wheelchair. Medicine (Baltimore). 2018;97(24).

16. Pousada T, Pereira-Loureiro J, Díez E, Groba B, Nieto-Riveiro L, Pazos A. Needs, demands and reality of people with neuromuscular disorders users of wheelchair. Examines Phys Med Rehabil [Internet]. 2017 Nov 13 [cited 2019 Jul 15];1(1). Available from: https://ruc.udc.es/dspace/handle/2183/20294

17. Enright P. The six-minute walk test. Respir Care. 2003;

18. Mylius C, Paap D, Takken T. Reference value for the 6-minute walk test in children and adolescents: a systematic review. Expert Rev Respir Med. 2016;

19. Peters D, Fritz S, Krotish D. Assessing the reliability and validity of a shorter walk test compared with the 10-Meter Walk Test for measurements of gait speed in healthy, older adults. J Geriatr Phys Ther. 2013;

20. Garcia C. Adaptación transcultural y validación de la versión en español de la Pediatric Balance Scale. Universidad de Extremadura; 2017.

21. Uniform Data System for Medical. Functional Independence Measure, version 5.1. New York: Buffalo General Hospital; 1997.

22. Martínez-Martín P, Fernández-Mayoralas G, Frades-Payo B, Rojo-Pérez F, Petidier R, Rodríguez-Rodríguez V, et al. Validación de la Escala de Independencia Funcional. Gac Sanit. 2009;23(1):49–54.
